# P01-036 – Systemic amyloidosis presenting with amyloidoma

**DOI:** 10.1186/1546-0096-11-S1-A40

**Published:** 2013-11-08

**Authors:** H Nalcacioglu, G Genc, S Ayyildiz, M Kefeli, O Aydin, M Elli, M Ceyhan, O Ozkaya

**Affiliations:** 1 Pediatric Nephrology, Ondokuz Mayis University Faculty of Medicine, Samsun, Turkey; 2 Pediatric Surgery, Ondokuz Mayis University Faculty of Medicine, Samsun, Turkey; 3 Pathology, Ondokuz Mayis University Faculty of Medicine, Samsun, Turkey; 4 Pediatric Oncology, Ondokuz Mayis University Faculty of Medicine, Samsun, Turkey; 5 Radiology, Ondokuz Mayis University Faculty of Medicine, Samsun, Turkey

## Introduction

Amyloidosis is a heterogeneous group of disorders characterized by extracellular deposition of unique protein fibrils. The least common presentation of an amyloid deposition is as a discrete mass called amyloidoma or amyloid tumor. It has been reported in many anatomic site including the respiratory, genitourinary, and gastrointestinal tracts, as well as internal viscera, the central nervous system, skin, breast, and soft tissues. We report a case of a soft tissue amyloidoma in the abdomen of an 16-year-old girl diagnosed with systemic amyloidosis.

## Case report

A 16-year-old girl was admitted to the hospital with the complaint of abdominal pain and artralgia for 4 months. She was referred to our hospital with a pre- diagnosis of a retroperitoneal mass documented with an abdominal ultrasonography and tomography. Her physical examination was normal except pretibial edema. Proteinuria, hypoalbuminemia, hypertriglyceridemia and nephrotic range proteinuria was found in laboratory examination. She underwent a surgery for complete resection of the lesion and routine histopathological examination with Congo red and crystal violet dyes verified the diagnosis of an amyloidoma. Immunohistochemical study for AA protein is positive. Nephrotic syndrome was diagnosed and renal biopsy was compatible with AA amyloidosis. A search for systemic disease was performed. Further investigations, for the etiology of the systemic amyloidosis;only heterozygous V726A was detected. Since the other causes of secondary amyloidosis were ruled out, the diagnosis of familial Mediterenean fever was made and treatment with colchicine and anakinra (1mg/kg/day sc) were started. After 3 months of the anakinra treatment, laboratory findings returned to normal and excessive proteinuria disappeared.

## Discussion

Amyloidoma is an unusual cause of soft tissue mass in the abdomen however a systematic approach incorporating clinical, radiological and pathological assessments will lead one to reach the diagnosis. Anakinra treatment is effective in the treatment of kidney and GIS amyloidosis.

## Disclosure of interest

None declared.
